# Global Imperative of Suicidal Ideation in 10 Countries Amid the COVID-19 Pandemic

**DOI:** 10.3389/fpsyt.2020.588781

**Published:** 2021-01-13

**Authors:** Teris Cheung, Simon Ching Lam, Paul Hong Lee, Yu Tao Xiang, Paul Siu Fai Yip, The International Research Collaboration on COVID-19

**Affiliations:** ^1^School of Nursing, The Hong Kong Polytechnic University, Hong Kong, China; ^2^Squina International Centre for Infection Control, The Hong Kong Polytechnic University, Hong Kong, China; ^3^Faculty of Health Sciences, University of Macau, Macau, China; ^4^Centre for Suicide Research and Prevention, University of Hong Kong, Hong Kong, China

**Keywords:** suicidal ideation, COVID-19, multi-country, mental health promotion, PHQ-9 = Patient Health Questionnaire

## Abstract

**Background:** The novel coronavirus (COVID-19) has had a detrimental impact on individuals' psychological well-being; however, a multi-country comparison on the prevalence of suicidal ideation due to the virus is still lacking.

**Objectives:** To examine the prevalence and correlates of suicidal ideation among the general population across 10 countries during the COVID-19 pandemic.

**Materials and methods:** This was a cross-sectional study which used convenience sampling and collected data by conducting an online survey. Participants were sourced from 10 Eastern and Western countries. The Patient Health Questionnaire (PHQ-9) was used to measure the outcome variable of suicidal ideation. Ordinal regression analysis was used to identify significant predictors associated with suicidal ideation.

**Results:** A total of 25,053 participants (22.7% male) were recruited. Results from the analysis showed that the UK and Brazil had the lowest odds of suicidal ideation compared to Macau (*p* < 0.05). Furthermore, younger age, male, married, and differences in health beliefs were significantly associated with suicidal ideation (*p* < 0.05).

**Conclusions:** The findings highlight the need for joint international collaboration to formulate effective suicide prevention strategies in a timely manner and the need to implement online mental health promotion platforms. In doing so, the potential global rising death rates by suicide during the pandemic can be reduced.

## Introduction

The 2019 novel coronavirus (COVID-19) was believed to have originated in Wuhan, China in late December 2020 and has rapidly spread to more than 200 countries nationwide (1). As of 4 June 2020, a total of 6,414,828 confirmed infected cases were recorded which resulted in 382,867 deaths (2). The global mortality rate was estimated to be 5.97%.

The rapid spread of COVID-19 has negatively affected the general population and some vulnerable subpopulations including the infected patients, their close contacts, frontline health professionals (3, 4), mentally ill individuals (5), and older adults (6, 7). The stringent infection control measures (e.g., quarantine, social distancing, lockdown, suspension of face-to-face teaching/learning in educational institutes) initiated by the health authorities in different countries were deemed effective in containing the virus; however, these stringent preventive measures also triggered negative psychological responses including fear of contagion, anxiety, uncertainty, posttraumatic stress symptoms (6), depression (8), and at worst, suicide. Furthermore, infected and suspected cases were prone to social stigma due to being placed in quarantine and how this has been negatively portrayed by the media (9).

Unfortunately, at the time of reporting, there were still no signs of effective vaccines in treating the infection, nor were there any evidence-based control measures to curb the rapid transmission of COVID-19. Consequently, the prevalence of suicide could reach a record high as the pandemic continues to spread across different countries, which may result in a suicide pandemic (10). This suicide pandemic could further emerge as most countries are confronted with a global economic crisis. To determine the prevalence of potential suicide, one can investigate SI as it has been shown to predict suicidal attempts and other risk-taking behaviors among all age groups (11). SI refers to having destructive thoughts and plans about dying. To determine the prevalence of potential suicide, one can investigate suicidal ideation, which refers to having destructive thoughts and plans about dying, as it has been shown to predict suicidal attempts and other risk-taking behaviors among all age groups.

Nonetheless, a global picture exploring the prevalence of suicidal ideation (SI) among the general population across different countries is still lacking. This gives us the impetus to fill this research gap through the conceptualization of the present study. To the best of our knowledge, this is the first multi-national cross-sectional observational study examining the prevalence of SI and its correlates among the general population.

The aims of this study were thus to (1) examine the overall prevalence of SI and its correlates across 10 countries in the East and West, (2) examine the association between individuals' health beliefs and suicidality, and (3) provide directions for the global imperative for suicide research and prevention. We had established three hypotheses: (1) the prevalence of SI among Asian countries in the East was higher than Western countries in this study; (2) gender was a significant covariate in predicting SI; and (3) health belief was significantly associated with face mask wearing and SI in this study.

## Materials and Methods

This was a cross-sectional observational study which used convenience sampling. An online survey hyperlink was distributed to the collaborative partners to disseminate to the general population in their respective countries / regions. The sampling frame was sourced from our collaborators in 10 countries / regions nationwide (the United States of America, Canada, the United Kingdom, Brazil, Philippines, Republic of Korea, China, Turkey, Hong Kong, and Macau).

### Participants, Inclusion, and Exclusion Criteria

This multi-national study targeted participants from the general population, and the eligibility criteria were: (1) aged 18 years or above; (2) male or female currently residing in a country affected by COVID-19; (3) able to read and / or understand Chinese, English, or the official language of their country of origin; and (4) capable of providing written consent. Participants who did not provide written consent and countries with <100 responses were excluded from this study.

### Data Collection

The questionnaire was disseminated via several online platforms including a discussion forum, community peer groups (e.g., COVID-19 information groups, child parenting groups, working adult peer groups), and organizational or personal Facebook pages. Data collection took place from 24 March to 30 April 2020.

### Measurement

Sociodemographic variables including gender, age, marital status, occupation (student or working-age adult), occupation (healthcare worker or non-healthcare worker) were solicited. Other variables including participants' frequency of using a face mask for self-protection and protecting others was also included [measured by the Face Mask Use Scale, FMUS (12)]. In addition, participants were also asked about their health beliefs which included beliefs about the susceptibility of being infected by the virus, severity toward the virus, cue to action by their governments / family members / friends, knowledge of COVID-19, and self-efficacy of wearing face masks correctly [items were applicable in the previous studies (13, 14)].

The Patient Health Questionnaire 9 (PHQ-9) was used to measure the outcome variable in this study. We used the English and Chinese version of the PHQ-9 in Western countries and Asian countries / regions, respectively. The Portuguese, Korean, and Turkish versions of the PHQ-9 were used according to the established and published version (15). The psychometric properties of the English version PHQ-9 showed satisfactory internal consistency (α = 0.83–0.92), convergent and discriminant validity, and construct validity (exploratory factor analysis and known-group method) (16).

The PHQ-9 consisted of nine items which measured the presence and severity of self-reported depressive symptoms in the 2 weeks prior to completing the questionnaire. Each item had a response range from 0 to 3, with a summed total score ranging from 0 to 27. A score of 5–9 indicated “mild” depression, 10–14 indicated “moderate” depression, 15–19 indicated “moderately severe” depression, and ≥ 20 indicated “severe” depression. Cronbach's alpha for the internal consistency reliability of the Chinese version of the PHQ-9 was 0.86 and the correlation coefficient for the 2-weeks test–retest reliability of the scale was 0.86 (17). Cronbach's alpha for the PHQ-9 in this study was 0.91. Three countries used the translated PHQ-9 (translated according to their native language). Backward and forward translations of the sociodemographic items from English into the official language were done for Brazil (Portuguese), Korea (Korean), and Turkey (Turkish), by the research team collaborators. Semantic equivalence and content validity were both established to ensure the appropriateness of the translations and relevance of the items.

### Bias

To reduce sampling bias and yield a representative sample, all countries described as having a “High risk” or “Low risk” (2) of contracting COVID-19 were invited to participate in this study.

### Sample Size

We aimed to recruit as many participants as possible over the recruitment period to improve the potential representativeness of the sample and thus did not calculate a minimum sample size a priori.

### Quantitative Variables

Since our study only focused on SI, the sum total score of question nine in the PHQ-9 (18) was extracted as a composite score of our dependent variable. Question nine consisted of questions about participants' thoughts, for example, that he / she would be better off dead, or of thinking of hurting themselves in the past 2 weeks. Participants indicated their answers on a 3-point scale, with “0:” no SI, “1:” SI for several days, “2” having SI for more than half the day, and “3” having SI nearly every day. Furthermore, higher scores indicated higher SI.

### Statistical Analysis

Data analyses were performed using SPSS 25.0 for Windows (SPSS Inc., Chicago, IL, USA). Descriptive analysis, chi-square statistics, and independent sample *t*-tests were used to examine the associations between sociodemographic characteristics, face mask use, core components of the Health Belief Model (HBM), and SI. Furthermore, ordinal regression analysis was performed to identify factors which were independently associated with SI. All the significant sociodemographic characteristics, face mask use patterns, and the HBM components were entered into the regression analysis as independent variables. The level of significance was set as *p* < 0.05 (two-tailed).

## Results

### Sociodemographic Characteristics

A total of 25,053 participants (77.3% female) were recruited, yielding a response rate of 52.8% in this study. Twelve countries participated, of which two countries (Finland and Sweden) had <100 participants and were thus excluded from analysis. This exclusion resulted in 10 countries being included in the statistical analysis (Table 1).

**Table 1:** Demographic characteristics of the participants and their associations with suicide ideation (*N* = 24,849).

	**Not at all**	**Several days**	**More than** **half the** **days**	**Nearly** **every day**
Overall	20,958 (84.3%)	2,627 (10.6%)	824 (3.3%)	440 (1.8%)
Country/region***				
USA	570 (79.5%)	79 (11.0%)	46 (6.4%)	22 (3.1%)
Canada	436 (85.8%)	51 (10.0%)	14 (2.8%)	7 (1.4%)
UK	781 (92.4%)	51 (6.0%)	7 (0.8%)	6 (0.7%)
Brazil	7,740 (92.4%)	430 (5.1%)	83 (1.0%)	122 (1.5%)
Philippines	341 (75.1%)	80 (17.6%)	19 (4.2%)	14 (3.1%)
Republic of Korea	590 (89.7%)	40 (6.1%)	19 (2.9%)	9 (1.4%)
China	812 (84.9%)	88 (9.2%)	38 (4.0%)	18 (1.9%)
Turkey	661 (84.5%)	67 (8.6%)	32 (4.1%)	22 (2.8%)
Hong Kong	8,866 (78.0%)	1,725 (15.2%)	562 (4.9%)	215 (1.9%)
Macau	161 (86.6%)	16 (8.6%)	4 (2.2%)	5 (2.7%)
Age***				
18–24	2,216 (78.2%)	371 (13.1%)	133 (4.7%)	112 (4.0%)
25–34	5,429 (81.2%)	817 (12.2%)	296 (4.4%)	148 (2.2%)
35–44	6,484 (84.5%)	853 (11.1%)	230 (3.0%)	102 (1.3%)
45–54	3,816 (87.4%)	379 (8.7%)	124 (2.8%)	49 (1.1%)
55–64	2,142 (92.8%)	134 (5.8%)	18 (0.8%)	14 (0.6%)
65+	559 (97.0%)	14 (2.4%)	3 (0.5%)	0 (0.0%)
**Sex**				
Male	4,538 (83.4%)	602 (11.1%)	205 (3.8%)	94 (1.7%)
Female	16,343 (84.6%)	2,010 (10.4%)	614 (3.2%)	342 (1.8%)
Marital status***				
With partner	7,821 (81.6%)	1,157 (12.1%)	378 (3.9%)	230 (2.4%)
Without partner	13,094 (86.1%)	146 (9.6%)	445 (2.9%)	210 (1.4%)
Occupation***				
Healthcare worker	6,289 (88.3%)	571 (8.0%)	153 (2.1%)	113 (1.6%)
Non-healthcare worker	14,669 (82.8%)	2,056 (11.6%)	671 (3.8%)	327 (1.8%)

* / ** / *** significant at 5% / 1% / 0.1% level.

The overall prevalence of SI ranged from 7.6 to 24.9% in our sample. Males exhibited higher levels of SI than females (16.6 vs. 15.4%). The youngest age group (18–24) showed the most prevalence for SI (21.8%) and there was an upward decreasing trend between age and SI. Furthermore, participants who originated from either the U.S. / Philippines accounted for the highest prevalence of suicidality (nearly every day; both 3.1%), followed by Turkey (2.7%), Macau (2.6%), China (1.9%), and Hong Kong (1.9%). Overall, UK participants showed the lowest prevalence of SI (0.7%), followed by South Korea / Canada (1.4%) and Brazil (1.5%).

### Prevalence of Suicidal Ideation in Different Nations in Relation to Demographic Characteristics

Table 1 showed the prevalence of SI and its association with demographic factors. A total of 3,891 (15.7%) participants had reported to have SI in the last 2 weeks. The prevalence across different regions was significantly different (*p* < 0.001), the lowest at 7.6% in the UK and the highest at 24.9% in the Philippines. Younger participants, those with a partner, and non-healthcare workers showed a higher prevalence of SI (*p* < 0.001).

### Association Between Face Mask Wearing, Health Belief, and Suicidal Ideation

Table 2 showed the association between face mask wearing, health belief, and SI. Participants without any SI were less likely to wear a face mask for self-protection / others as they perceived themselves as being less susceptible to contracting COVID-19 and perceived this novel virus as not being severe, although they had been given cues to wear face masks and had good knowledge about COVID-19.

**Table 2:** Face-mask wearing behavior, health belief, and their associations with suicide ideation (*N* = 24,849).

	**Not at all**	**Several days**	**More than** **half of** **the days**	**Nearly** **every day**
**Face Mask Use (FMUS)**				
Self-protection***	7.56 (3.56)	8.08 (3.09)	8.20 (3.05)	7.70 (3.74)
Protecting Others***	7.49 (4.14)	8.30 (3.48)	8.46 (3.34)	7.73 (4.04)
Total***	15.10 (7.34)	16.40 (6.24)	16.67 (6.12)	15.47 (7.46)
**HBM**				
Susceptibility***	3.22 (1.22)	3.23 (1.11)	3.28 (1.02)	3.56 (1.22)
Severity***	5.89 (1.61)	6.45 (1.48)	6.59 (1.51)	6.68 (1.54)
Cue***	13.90 (2.19)	13.47 (1.93)	13.18 (1.99)	13.12 (2.18)
Knowledge***	5.42 (1.20)	5.15 (1.14)	5.13 (1.20)	5.11 (1.49)
Efficacy	3.13 (0.76)	3.12 (0.66)	3.16 (0.70)	3.13 (0.81)
Total	31.56 (3.87)	31.42 (3.35)	31.33 (3.54)	31.60 (3.88)

* / ** / *** significant at 5% / 1% / 0.1% level; HBM: Health Belief Model.

### Significant Predictors Associated With Suicidal Ideation Using Ordinal Regression Analysis

Results of the ordinal regression (Table 3) confirmed the univariate comparison results and showed that relative to Macau, participants in the UK and Brazil had less frequent SI (OR = 0.38 and 0.31, respectively, *p* < 0.001). Younger, male, and participants with a partner showed higher levels of SI (all *p*'s < 0.05). Furthermore, face mask wearing was not associated with SI. Participants who believed that they were susceptible to the disease, perceived the disease as severe but had a low cue to action, poor knowledge of the disease, and poor efficacy to wear a mask properly, exhibited higher levels of SI (*p* < 0.001).

**Table 3:** Ordinal regression on suicide ideation in the past 2 weeks (*n* = 24,849).

	**Odds ratio (95% CI)**	***p*-value**
**Country/Region**		
USA	0.94 (0.58, 1.53)	0.81
Canada	0.76 (0.45, 1.27)	0.29
UK	0.39 (0.23, 0.67)	**0.001**
Brazil	0.32 (0.20, 0.50)	**<0.001**
Philippines	1.18 (0.72, 1.94)	0.51
Republic of Korea	0.72 (0.44, 1.21)	0.22
China	1.25 (0.78, 2.01)	0.36
Turkey	0.84 (0.52, 1.37)	0.49
Hong Kong	1.50 (0.96, 2.34)	**0.08**
Macau	Ref	
**Age**		
18–24	5.98 (3.56, 10.03)	**<0.001**
25–34	3.96 (2.38, 6.61)	**<0.001**
35–44	3.05 (1.83, 5.09)	**<0.001**
45–54	2.68 (1.60, 4.49)	**<0.001**
55–64	1.84 (1.08, 3.14)	**<0.001**
65+	Ref	
**Sex**		
Male	1.16 (1.06, 1.27)	**0.001**
Female	Ref	
**Marital status**		
With partner	0.70 (0.65, 0.76)	**<0.001**
Without partner	Ref	
**Occupation**		
Healthcare worker	1.03 (0.93, 1.14)	0.55
Non-healthcare worker	Ref	
**Face Mask Use**		
Self-protection	1.01 (0.99, 1.03)	0.21
Protecting Others	1.00 (0.98, 1.01)	0.60
**Health Belief Model**		
Susceptibility	1.17 (1.12, 1.21)	**<0.001**
Sever	1.15 (1.12, 1.19)	**<0.001**
Cue	0.86 (0.84, 0.88)	**<0.001**
Knowledge	0.92 (0.89, 0.95)	**<0.001**
Efficacy	0.88 (0.83, 0.93)	**<0.001**

*Bold for P-value significant at < 0.05*.

## Discussion

### Age

Our results showed that the youngest age group (18–24 years) had the highest odds (OR = 5.98 95% CI 3.56, 10.03) of harboring SI compared to the oldest group (≥60 years old). The younger the age, the higher the likelihood of reporting SI; thus, younger age seemed to be a significant factor associated with SI. Consistent with this finding, some researchers (19) recently reported the association between COVID-19 and youth mental health. They found that 40.4% of the sampled youth (*n* = 584; 14–35 years old) were prone to psychological problems, of whom, 14.4% exhibited signs of posttraumatic stress symptoms. The high prevalence of these symptoms suggests that COVID-19 infection has a significant influence on youth mental health.

Due to suspension of face-to-face teaching in all education institutions during the pandemic, students have had to shift to online modes of learning. This sudden shift of their traditional learning mode may have led to maladaptation and increased levels of stress, anxiety, and academic pressure. In addition, students may have received less peer support due to a reduction in face-to-face interactions, being deprived of extracurricular activities (e.g., sports, gymnasium, youth centers) and entertainment (e.g., cinemas, theme parks, playground) due to lockdown measures. The full-scale lockdown measures in some regions / countries (e.g., China, Hong Kong, Brazil, the UK, the U.S., Italy) may have led to increased levels of social isolation, entrapment, and loneliness; consequently, contributing to elevated risk of SI among the youth (20). Cultural and demographic characteristics may have also contributed to SI in the youth, as noted by Khan et al. (21) that male students from low-income families reported higher levels of academic stress and SI. Furthermore, other researchers (22) examined the association between physical activity and sedentary behavior among adolescent's with suicidal vulnerability. Using the Global School-based Student Health Survey data from 206,357 students (mean age 14.6 ± 1.18 years; 51% female) in 52 low-and-middle income countries, results showed that students with high leisure activity and low sedentary behavior (≥3 h/day) was independently associated with higher odds of SI, whereas insufficient physical activity and high sedentary behavior was associated with higher odds of SI for both male and female adolescents.

Findings from another cross-sectional study conducted by Ahmed et al. (23) in Hubei, the epicenter of the coronavirus, found that young adults aged between 21 and 40 years had higher rates of anxiety, depression, alcohol use, and lower mental well-being during the COVID-19 pandemic. In fact, previous epidemiological studies found an association between psychiatric symptoms and suicidal tendency among survivors of the 2003 SARS epidemic (24). In other words, it has been consistently found that there is a positive correlation between infectious disease epidemics, psychiatric symptoms, and suicidal tendencies. Since the COVID-19 pandemic presents an unprecedented global public health risk, individuals residing in countries affected by this pandemic are overwhelmed with an overflow of information on the latest public health control measures to mitigate the impact of rapid transmission of this novel virus. This overflow of information and the relaying of fake news has inevitably created the “infodemic” on social media which triggers a panic state leading to SI or suicidal behavior (25). Non-schoolers / working age youth, on the other hand, may worry about their future career prospects in light of the imminent global economic recession brought on by this COVID-19 pandemic.

### Gender

It was found that being male was one of the significant factors associated with SI. Compared to women, men were less likely to seek social, emotional, or professional support / counseling from others during the crisis, partly due to their hegemonic masculine beliefs (26). Due to the pandemic, several male participants were confronted with sudden loss of employment which led to increased financial burden (27) and marital discord. These male, unemployed individuals may have associated their job loss with their diminished functional role as a breadwinner in the family. Furthermore, they may have subjectively felt less valued / important by their family members and perceived themselves as a burden to the family. Furthermore, the present study found that relationship crisis, increased interfamilial conflict, financial burden, and weakened masculine identity may also have triggered SI among male participants.

### Marriage

Marital status has been identified as a risk factor for suicide in mental health research (28). This is because marriage serves a protective function by providing social support, facilitating social participation, and increasing self-esteem (29). Furthermore, marriage is associated with larger social networks (30), an increased sense of belonging (31), promoting well-being, and as a buffer against mental illness. Moreover, the Interpersonal Theory of Suicide (32) posits that the sense of belonging may also explain the relationship between marital status and suicide. This theory proposes that thwarted belongingness and perceived burdensomeness contribute to suicidal desire and suggests that harboring could be an independent predictor of SI for men (33). Among all the factors associated with SI, financial hardship was one of the strongest predictors of SI (34). Furthermore, parents' abusive / volatile behavior may indirectly be associated with adolescents' SI (35). Individuals who encountered a sudden loss of unemployment / redundancy may experience acute stress, anxiety, uncertainty, and feel as if they are a financial and psychological burden on their family members. Thus, governments are playing a pivotal role by providing timely financial safety nets (e.g., food, shelter, financial subsidy, unemployment subsidy) (10) to the vulnerable population to reduce the increased likelihood of psychiatric symptoms including post-traumatic stress disorder (PTSD), depression, and SI. Consistent with our findings, later research such as Miret et al. (36) asserted that marital status and occupational status were associated with lifetime SI in individuals aged between 18 and 49.

### Health Belief

Results from the ordinal regression analysis showed that all the core components of the HBM (susceptibility, severity, cues to action, knowledge, and efficacy) were significant predictors of SI in this study (Table 3). Thus, this suggests that participants' health belief is a critical factor in determining an individual's SI. Although there was some empirical evidence supporting HBM in the domain of physical health (37), there was insufficient evidence to illustrate that health belief can also influence mental health (38). Furthermore, health belief is often linked to an individual's health literacy (39); therefore, in any pandemic, accurate and up-to-date health information should be disseminated by risk communicators of the health authority to the general population to allay public fear and anxiety. For instance, public health education on preventive measures (frequent handwashing/hand hygiene, social distancing, avoid overcrowding, face mask wearing for self-protection/ others) should be disseminated via digital online platforms, mass media, and news reports to improve the health literacy of the public. Consequently, individuals with higher levels of health literacy will improve their health behavior which is crucial to prevent the rapid transmission of the virus in the wider community. Furthermore, these individuals will take primary responsibility, action, and behavior to protect themselves / others in their primary setting to combat against the pandemic (40, 41).

It is evident that the governments of different nations have adopted different public health preventive strategies to mitigate against the rapid transmission of COVID-19. The significant variation in the mortality rate and number of suspected / confirmed COVID-19 patients between different countries / regions may reflect the determination and effectiveness of these infection control initiatives by the governments / health authorities. Inevitably, the COVID-19 pandemic has negatively impacted individual's mental health including perceived loneliness, social isolation, boredom, decreased quality of life, fear, anxiety, uncertainty, depressive symptoms, posttraumatic stress symptoms, and SI. Furthermore, the increase in suicide during the quarantine is not inevitable if appropriate measures were in place (42).

### Regional Differences in Suicidal Ideation

Compared to Macau, participants in the UK and Brazil reported less SI. It is surprising to note that the UK and Brazil had lower odds of SI than that of Macau (Table 3). We speculate two reasons that could have explained this result. Firstly, Macau is a well-known country for migrant workers who have been suffering social inequities such as social isolation, overcrowded living conditions, lack of access to sanitizers, and personal protective equipment (43). They may also have difficulty obtaining health care compensation (44) and may not fully understand the pandemic situation due to language barriers and poor health literacy (45). In addition, these migrants may not be eligible to apply for the Macau government's financial subsidy and welfare benefits during this pandemic; thus, migrants in Macau may be a part of the vulnerable subpopulation who have an increased tendency to experience mental health problems which leads to increased odds of SI.

Therefore, essential COVID-19 related health information should be translated and disseminated in different languages to allay these migrants' anxiety and fear. Secondly, the variation in SI across different countries can be explained by the total government expenditure and financial resources available for their citizens. To illustrate, participants who originated from low-income countries such as Brazil and Philippines may have received limited welfare support from their governments compared to those residing in middle-to-high income countries (e.g., Hong Kong, U.S., UK, and Macau). Therefore, economic adversity alongside poor welfare support and medical benefits may increase the odds of having SI due to a lack of hope for the future during the pandemic.

Our results have shed important insights into mental / public health research during the COVID-19 pandemic and has shown that there seems to be a significant statistical difference between participants with / without SI between Macau (the East) and the UK and Brazil (the West). The variation in our prevalence estimates is likely to be attributed to different socio-cultural and economic contexts, and public health strategies in infection control, alongside government's efforts to help reduce the impact compounded by the pandemic through financial subsidy, preventive measures (mask supply, personal protective equipment), and other practical support provided to the community (10).

Despite a concerted effort across the globe, evidence from older research findings suggested that the U.S. had increased rates of suicide during the influenza pandemic between 1918 and 1919 (46) and among older adults during the 2003 SARS epidemic (5, 47). This trend is somewhat changing with youth suicide becoming more prevalent in the last few decades (48). Findings emerging from this study have provided scientific evidence that younger adults seem to have an elevated risk of suicide than older adults. Furthermore, our key findings have highlighted that there is a pressing need for implementation of contingent global mental health prevention measures and interventions for the community at large, specifically in the vulnerable subpopulations (10), *via* joint international collaboration (49). In addition, depression was found to be closely linked to suicide (50). Although depression is a treatable psychiatric disorder, it needs to be treated in a timely fashion before leading to SI.

Suicide is a complex and multifaceted phenomenon. It is evident that the COVID-19 pandemic has impacted different work sectors (27) with social distancing / quarantine measures possibly inducing loneliness, fear, anxiety, and withdrawal in some vulnerable individuals across all age groups (51).

### Implications of the Study

Therefore, the WHO should establish a global mental health crisis team chaired by public health / mental health experts and epidemiologists from multidisciplinary backgrounds to establish and deliver three-tiered telehealth, telemedicine, and remote psychological counseling. With the aid of advanced digital technology, health experts can identify the most at-risk groups in the community and offer imminent assistance to reduce the global mortality burden caused by suicide. Self-guided digital interventions should be widely promoted, especially in some nations where there is restricted access to traditional health services due to lockdown / quarantine (52). Additionally, hotlines and text line support would be viable tools to mitigate against the risk of isolation and anxiety (10, 42). Furthermore, on-site visits by psychiatrists / psychologists are warranted to provide timely treatment to individuals with elevated risks of suicide.

### Limitations / Generalisability

There are several limitations that need to be addressed in this study. First, a cross-sectional study cannot infer any causal relationship between variables and thus, may reduce the generalisability of our findings. Second, some countries with a high or low risk of COVID-19 infections could not participate in this study due to the lengthy ethical approval process which inevitably reduced the representativeness of our sample. Third, some variables (monthly household income, educational attainment, number of children) were not solicited in this study; thus, we could not examine the association between these demographic characteristics with SI. Lastly, owing to the sudden surge of the pandemic emerging in the East before its rapid spread to the Western countries, use of stratified random sampling was almost impossible in this multi-national collaborative study. Therefore, future researchers should consider these factors when investigating the impact of SI and COVID-19 in future studies.'

## Conclusion

This study found that SI was prevalent among the younger age groups as well as in male, married individuals across all the collaborative countries / regions. Furthermore, health belief was significantly associated with SI during the COVID-19 pandemic; thus, joint international collaboration by key stakeholders and policymakers is warranted to formulate timely and effective suicide prevention measures and mental health promotion initiatives. In doing so, the global risk of increasing psychiatric morbidity and rising mortality rates brought on by this pandemic can be reduced.

## Data Availability Statement

The raw data supporting the conclusions of this article will be made available by the authors, without undue reservation.

## Ethics Statement

The studies involving human participants were reviewed and approved by several regional committees, including Human Subjects Ethics Sub-committee of the Hong Kong Polytechnic University (reference no: HSEARS20200227002-01), the National Committee of Ethics Research (CONEP) (CAAE: 30572120.0.0000.0008; Opinion 3.971.512), Medical Sciences Interdivisional Research Ethics Committee, University of Oxford (R69446/RE001), Delegated Research Ethics Review Committee, and York University (File # 2020103). The patients/participants provided their written informed consent to participate in this study.

## The International Research Collaboration on COVID-19

- Dr. Lorna Kwai Ping Suen, BN, MPH, PhD, Squina International Center for Infection Control, School of Nursing, The Hong Kong Polytechnic University, Hong Kong. Email: lorna.suen@polyu.edu.hk. ORCID: 0000-0002-0126-6674
- Ms. Hilda Sze Wing Ho, MA, MPH, PhD (Candidate), Department of Psychology, York University, Canada. Email: hildaho@yorku.ca. ORCID: 0000-0002-7966-4285
- Dr. Kin Bong Hubert Lam, PhD, Nuffield Department of Population Health, University of Oxford, United Kingdom. Email: hubert.lam@ndph.ox.ac.uk. ORCID: 0000-0003-1228-3362
- Dr. Emma Yun-zhi Huang, DPH, MPH, Division of Pre-school Education, Zhongshan Polytechnic, Zhongshan City, Guangdong Province, PRC. E-mail: huangyunzhiemma@sina.com. ORCID: 0000-0001-5967-2731
- Prof. Ying XIAO, PhD, Faculty of Medicine, Macau University of Science and Technology, Macau. Email: yxiao@must.edu.mo
- Dr. Fernanda Maria Vieira Pereira-Ávila, PhD, Fluminense Federal University, Rio das Ostras, Brazil. Email: fernandamvp@id.uff.br. ORCID: 0000-0003-1060-6754
- Prof. Elucir Gir, PhD, University of São Paulo, School of Nursing at Ribeirão Preto, Brazil. Email: egir@eerp.usp.br. ORCID: 0000-0002-3757-4900
- Dr. Menevse Yildirim, PhD, Department of Nursing Management, Fethiye Faculty of Health Sciences, Muğla Sıtkı Kocman University, Mugla, Turkey. Email: menevsesamur@gmail.com; menevseyildirim@mu.edu.tr. ORCID: 0000-0001-6033-6196
- Prof. Seyda Seren Intepeler, PhD, Department of Nursing Management, Faculty of Nursing, Dokuz Eylul University, Izmir, Turkey. Email: seydaseren@gmail.com. ORCID: 0000-0001-8615-9765
- Dr. Tella Lantta, RN, PhD, Department of Nursing Science, University of Turku, Finland. Email: tella.lantta@utu.fi. ORCID: 0000-0001-7715-7573
- Dr. Kyungmi Lee, PhD, Samsung Medical Center, Seoul, Korea. Email: kyungmi79.lee@samsung.com. ORCID: 0000-0001-8615-9765
- Dr. Nayeon Shin, PhD. CHA University, Bundang CHA Medical Center, Gyeonggido, Korea. Email: nabong78@hanmail.net
- Mr. Laurence Lloyd Parial, MSc, School of Nursing, The Hong Kong Polytechnic University. Email: laurence.parial@connect.polyu.hk
- Mr. Tor Michael Rossing, MA, SAG Flowmedik Oy, Helsinki, Finland. Email: michael.rossing@flowmedik.com
- Ms. Ching Yuk Hon, BN, School of Nursing, The Hong Kong Polytechnic University. Email: ching-yuk.hon@connect.polyu.hk
- Ms. Merissa Tsang, MSc, Agape Acupuncture Clinic, San Mateo, California, USA. Email: merissa.tsang@gmail.com
- Ms. Jessica P. Braz Poeys, BSN, Westways Staffing Inc., Austin, USA. Email: jessicapb@hotmail.com. ORCID: 0000-0002-6431-6288
- Mr. Tommy Kwan Hin Fong, BA, MPsyMed School of Nursing, The Hong Kong Polytechnic University, Hong Kong SAR. Email: kfonguos@gmail.com
- Ms. Shun Chan, BN, RN, Squina International Centre for Infection Control, School of Nursing, The Hong Kong Polytechnic University, Hong Kong. Email: csschan2017@gmail.com.

## Author Contributions

SL and TC: conception and design of the study and drafting the manuscript. SL and the International Research Collaboration on COVID-19: acquisition of data. SL, PL, TC, and the International Research Collaboration on COVID-19: data analysis. SL, PL, and TC: interpretation of data. YX and PY: critically review. All authors contributed to the article and approved the submitted version.

## Conflict of Interest

The authors declare that the research was conducted in the absence of any commercial or financial relationships that could be construed as a potential conflict of interest.
